# A novel design for biodiesel production from methanol + mutton bone fat mixture

**DOI:** 10.1186/s13068-022-02229-4

**Published:** 2022-11-24

**Authors:** Ali Farokhnia, Seyyed Mohammad Jokar, Payam Parvasi, Albert S. Kim

**Affiliations:** 1 Department of Chemical, Petroleum and Gas Engineering, Shiraz University of Technology, Shiraz, Iran; 2 Civil and Environmental Engineering, University of Hawaii at Manoa, 2540 Dole Street, Holmes Hall 383, Honolulu, HI 96822 USA

**Keywords:** Methanol, Mutton bone fat oil, Biodiesel, Ultrasonic, Membrane filtration

## Abstract

Bioenergy plays a significant role in the green transition. In this work, the conversion of methanol and mutton bone fat oil (as a low-cost feedstock) for bioenergy production was studied. The five-level, three-factor response surface methodology (RSM) was used to optimize the transesterification reaction conditions for produced biodiesel. Twenty ultrasonic-assisted experiments at the frequency of 25 kHz were conducted to investigate the effects of methanol/oil molar ratio (M/O) and concentrations of KOH and NaOH as catalysts on biodiesel yield. A second-order polynomial equation was developed by fitting the RSM experimental data using Design-Expert software. Results showed that the optimum biodiesel yield of 90.087% could be achieved by the KOH catalyst with 2.5 *wt*% concentration and 15:1 M/O during 3 h of the reaction. Furthermore, the biofuel analyses showed that methanol and mutton bone fat oil can be used as a proper feedstock for biofuel production. In the following, a membrane filtration package system is proposed and modeled. The reaction kinetics was determined based on experimental data. The results of the mathematical modeling showed the reaction time appears to be 6 times shorter in a membrane setup (30 min). Consequently, membrane application is highly recommended for biodiesel production from mutton bone fat oil.

## Introduction

The demand for new energy sources is rising fast, because of rapid population growth and transportation networks. Therefore, one of the main issues for humanity in the current century is providing energy and natural sources being depleted [1]. It is predicted that by 2030, 80% of energy sources will comprise fossil fuels [2], which can last only a few decades in the twenty-first century. For example, diesel is a fossil fuel that causes many environmental problems due to releasing toxic compounds such as carbon monoxide, sulfur dioxide and nitrogen dioxide [2].

Biodiesel (monoalkyl ester) is one of the diesel-alternatives with less contamination potential, which can be obtained from renewable or natural resources [3]. Compared to fossil fuels, biodiesel has similar chemical properties to petroleum–diesel and emits fewer greenhouse gases and air-pertinent pollutants [4]. However, biodiesel is denser and more viscous with a higher cetane number than fossil diesel [5]. Biodiesels have high flash points and therefore they are inflammable [6]. Mixing biodiesel with diesel fuel leads to a decrease in unburned and polycyclic aromatic hydrocarbons released from diesel engines [7].

Methanol is the most frequently applied alcohol to achieve transesterification of biofuel production due to its low cost, physical and chemical advantages. It is as a key component of biodiesel and makes up about 20% of the biodiesel weight. Methanol as a fuel additive also can be blended with biodiesel in a car engine. A variety of edible oils could be used as feedstocks for biodiesel production (for example, rapeseed, sunflower, soybean, and palm oil). Still, it is not economically feasible to use edible oils for fuel. Thus, recent research has focused on non-edible oils, wastes of cooking oils, and waste animal fats as new alternatives for biodiesel production [8–11]. The volume of oil-free fatty acid (FA) has a direct impact on the biodiesel production process. When FA concentration is higher than 3% by weight, soap is formed during the production process and the catalytic activity decreases, and therefore the biodiesel generation efficiency is reduced [12, 13]. For high free fatty acids feedstocks, a two-step esterification–transesterification mechanism is often employed to improve the quality of biodiesel [14].

Vicente et al. [15] produced biodiesel from sunflower oil by the transesterification method using various catalysts. Because of the low free-FA content of the sunflower oil, biodiesel is generated by using only a one-step in situ transesterification reaction. In their study, biodiesel synthesis was carried out at 65 °C, the methanol to sunflower oil molar ratio of 6:1 and the catalyst concentration 1 *wt*% by weight in 4 h.

Wyatt et al. [16] have reported biofuel production from beef fat, chicken fat, and pork fat. They showed that the animal fats were featured with higher lubrication and oxidation stability as well as lower NOx content compared to soybean oil. Besides, the production of biodiesel from waste animal fat in the presence of methanol was experimentally examined in a work prepared by Srinivasan et al. [17]: the maximum yield of 94% was reported at the optimal conditions (M/O of 6:1, 0.5*wt*% catalyst, 60 °C, and 2 h).

Sonochemistry or ultrasound has been proven as a proficient way to increase the rate of chemical synthesis [18–23]. As the findings have shown, ultrasonic mixing can yield smaller droplets compared to a conventional mixer and therefore generates a much larger surface area available for the reaction process [24]. Normal-chain alcohols have a faster reaction rate, compared to the secondary and tertiary alcohols with an alcohol-to-oil moral ratio of 6:1. The low-frequency ultrasonic (28–40 kHz) is an effective way to lower the reaction time (10–20 min). Higher efficiency, but longer reaction time was obtained from the 28-kHz frequency [25].

Various vegetable oils, such as palm, soybean and sunflower oils, were studied for biodiesel production by Stavarache et al. [23]. The transesterification reactions were conducted using conventional and 45 kHz frequency ultrasound-assisted methods at 38 °C to 40 °C, ultrasonic waves are found to increase reaction efficiency and lower the reaction temperature.

Kelkar et al. [26] examined the effects of ultrasonic cavitation on the esterification process. The reaction between fatty acid and methanol took place in an ultrasonic system by the H_2_SO_4_ catalyst. The authors argued that ultrasonic waves increased biodiesel yield in comparison to conventional methods and lowered the reaction time. At optimized conditions (alcohol-to-oil molar ratio of 10:1, H_2_SO_4_ catalyst concentration of 2 *wt*%, reaction temperature of 40 °C and reaction time of 90 min), a yield of over 95% was obtained.

The soybean oil biodiesel production by ultrasonic waves was conducted by Naresh et al. [27], who obtained a biodiesel yield of 90% by using 0.5 *wt*% KOH catalyst concentration, 6:1 methanol:oil molar ratio, 611 kHz ultrasonic frequency and 30 min reaction time. The results showed that by high-frequency ultrasound more than 90% reaction yield was obtained in 30 min.

Deng et al. [28] worked on the Jatropha oil biodiesel production. An appropriate physical property of the product was reported by applying a two-step process using mechanical stirring at 600 rpm and ultrasound-assisted methods via the H_2_SO_4_ catalyst and transesterification with the NaOH catalyst. The results of the two-stage reaction showed that during 1 h reaction time of the esterification process and 30 min of the transesterification stage, the yield of biodiesel reached 96.4%. In addition, this research indicates the two-step process using ultrasonic system is the most effective method to produce biodiesel from high free fatty acid oils.

Gole and Gogate [29] investigated the application of ultrasonic devices for biodiesel generation from non-edible oils. The reaction rate was examined in a temperature change from 30 °C to 50 °C and a frequency of 20 kHz. They optimized the process by changing parameters such as alcohol/oil ratio, catalyst concentration, reaction time, and temperature. The optimal conditions for biodiesel synthesis in their research are: alcohol-to-oil molar ratio of 6:1, KOH catalyst concentration of 1 *wt*%, transesterification reaction temperature and time of 40 °C and 40 min, respectively.

Choudhury et al. [30], studied the ultrasonic-assisted biodiesel manufacturing from Jatropha oil. The results showed the highest transesterification efficiency is obtained by the alcohol-to-oil molar ratio of 11:1, the catalyst concentration is 5.5 *wt*% and the reaction temperature of 64 °C. Furthermore, by applying the ultrasonic method, it is possible to reduce the required energy for biodiesel synthesis by 20% compared to the conventional method.

Khan et al. [31] examined the biodiesel synthesis from Eucalyptus oil by the sonication system at various frequencies, powers, and temperatures. The maximum efficiency of 96.73% was achieved by the ultrasonic power of 110.25 W, alcohol/oil molar ratio of 6.36:1, a frequency of 29.54 kHz and reaction temperature and time of 35 °C and 8 min, respectively.

The chicken fat oil is used as a feedstock for producing biodiesel in an ultrasound-assisted system prepared by Fayyazi et al. [32]. The results showed that the yield of biodiesel production from chicken fat is 94.8% by 24 kHz ultrasonic frequency, the catalyst concentration of 1 *wt*%, alcohol-to-oil molar ratio of 7:1: and the reaction time of 9 min. In their work, the reaction time was reduced by 87.5% in the ultrasonic method in comparison to the conventional method.

Mirab et al. [33] studied the effect of ultrasound on biodiesel production from chicken feet oil. The results showed that using the methanol to oil molar ratio of 12:1, the KOH catalyst concentration of 1 *wt*% and the 45-kHz ultrasound frequency, the highest efficiency of 89.74% can be achieved.

In other study, a two-step esterification and transesterification process of diseased swine fat based biodiesel production was tested by ultrasound method [34]. The results showed that the transesterification reaction optimal condition are catalyst concentration 1.11 *wt*%, reaction temperature 62.3 °C, methanol/oil molar ratio 7.42:1 and reaction time 116.14 min. In their study, the catalyst concentration consumption was reduced by 63.3% in comparison with the previous works.

Nevertheless, conventional methods for biodiesel production have some disadvantages, such as the high methanol consumption for reversible transesterification [35, 36]. This will lead to enhanced reaction time as well as biodiesel production cost. Other drawbacks include the significant loss of methanol and water unreacted during biodiesel purification [37]. To overcome these problems, the membrane technique is suggested as an appropriate modification method by researchers over the last decade [36].

Membranes are widely used to separate a desirable or undesirable substances from a mixture (e.g., hydrogen, CO_2_, CH_4_, H_2_S, etc.) from gas mixtures [38–41]. The membrane separation process can remove the biodiesel from products after reacting in the reactor. The residue could be recycled back to the reactor for further reactions. On the other hand, the membrane dry-washing procedure could reduce water consumption and offer high fuel quality [42–44].

Sokac et al. [37] worked on polymeric membranes for biodiesel purification. The membranes include polyethersulfone, polyacrylonitrile, polypropylene and regenerated cellulose. They showed that polyacrylonitrile membrane could have a good performance for separating biodiesel produced by lipase-catalyzed transesterification. Alves et al. [44] used a 30-kDa membrane for biodiesel purification and indicated that the membrane technology is a suitable alternative for biodiesel purification. They approved that membrane technology is a suitable alternative for biodiesel purification [45].

Poly (ether sulfone) hollow fiber membranes (PES-HFM) were selected as a membrane in a work prepared by Noriega [46, 47] and the process was experimentally tested and modeled. Results showed that high-quality biodiesel could be obtained in a membrane reactor, generating 10 times better purity than that of the conventional reactor [47].

Cao et al. [48, 49] used a Filtanium ceramic membrane for canola oil biodiesel purification. By recycling the retentate to the reactor, high purity biodiesel was produced in their work. They also modeled the process and evaluated the reaction rate constants [50], considering NaOH catalyst with different weight percent (0.05, 0.1, and 0.5 *wt* %) and a methanol/oil ratio of 24:1 was used in their work. They showed that membrane technology enhances the reaction rate.

Talaghat et al. [51] modeled a continuous membrane tubular reactor used for biodiesel generation in the presence of an alkaline catalyst, comparing membrane and conventional reactors. The highest conversion was achieved in a membrane reactor with a methanol/oil ratio of 24.

In this work, the mutton bone oil, for the first time, was used as a raw material for producing biodiesel. Attempts have been made to accelerate the reaction rate by simultaneous application of an ultrasonic bath and magnetic stirrer. An experimental setup is designed to evaluate the impact of the alkaline catalysts (NaOH and KOH) in the transesterification process. The various variables (alkali catalysts, catalyst concentration and M/O) were optimized by using a central composite design (CCD) approach-based response surface methodology (RSM) to maximize the biodiesel yield with the aid of Design-Expert software. Finally, a mathematical model of biodiesel production in a membrane system is developed, and the reaction constants are calculated.

## Materials and methods

### Raw materials

The mutton bones were heated in water (1:3) at 80 °C for 4 h. The melted bone fat was collected from the water surface and centrifuged for 20 min to accumulate the oil. The methanol (99.85%) catalysts (KOH and NaOH) were supplied by Merck Company. The other used chemicals were analytical grade and obtained from Merck, Samchun, Carlo Erba, Scharlau, Alfa Aesar and Sigma-Aldrich.

### Experimental setup

The reaction was conducted in a 500-ml three-neck lab glass flask with a reflux condenser, a mechanical mixer (TAT, 1500 rpm), and a digital thermometer (TFA, 30.1048) submerged in an ultrasonic bath (Elma Ultrasonic TI-H-5 type, Germany, 3.5L, 25.45 kHz, and 100 W). The tests were conducted under atmospheric pressure.

### Transesterification process

The three-neck glass flask was filled with methanol and an alkali catalyst (KOH or NaOH). The flask was fixed in the ultrasonic cleaning bath and the mutton oil was poured into the flask. The reaction continued for 3 h at 60ºC with the frequency of 25 kHz and under mechanical stirring at 500 rpm. After timing the transesterification reaction, the glycerol and biodiesel layers were separated by centrifugation (200 rpm, 10 min). The mixture was settled for 12 h and the biodiesel layer was washed several times with distilled water to subtract the remaining catalyst, methanol, and soap. Afterward, the washed biodiesel was heated (107 °C, 1 h) to eliminate the remaining water and methanol. The following formula was applied to identify the biodiesel yield [52]:

1 $$Yield\left(\text{\%}\right)=\frac{{W}_{biodiesel}}{{W}_{mutton-oil}}\times 100,$$

where *W*_*biodisel*_ and *W*_*mutton-oil*_ are the weight of biodiesel and the weight of mutton oil, respectively.

### Experimental design

The RSM was applied to examine the impacts of catalyst type, catalyst concentration, and M/O on the transesterification process. The numerical form of independent variables can be created as Eq. (2) [53]:

2 $$Y=F\left({X}_{1},{X}_{2},{X}_{3},\dots ,{X}_{n}\right)\pm c,$$

where Y is the response (e.g., the yield), F stands for the function, c stands for the experimental deviation, and X_1_,X_2_,X_3_, …,X_n_ represent independent variables.

If the response (y) is matched by a linear function of the independent variable, Eq. (2) could be rewritten as Eq. (3):

3 $$ Y = \beta_{0} + \beta_{1} X_{1} + \beta_{2} X_{2} + \cdots + \beta_{n} X_{n} \pm c. $$

Here, the type of catalyst was represented as a discrete variable and the M/O as well as the catalyst concentration were considered the continuous variables. The continuous variables were selected in 5 levels. A mathematical equation can be written using an RSM of 20 experiments. Table 1 lists the coded and not-coded independent variables (Xi) and levels for CCD applied to Design-Expert software.

**Table 1:** Factors and their levels for central composite design

Variable	Symbol	Coded factor level				
		− 2	− 1	0	1	2
M/O	X_1_	6	9	12	15	18
Catalyst amount (*wt*%)	X_2_	1	1.5	2	2.5	3
Catalyst type	X_3_		NaOH		KOH	

The experimental data were analyzed by the second-order polynomial regression model (Eq. (4)) [54]:

4 $$ Y = \beta_{0} + \,\sum\nolimits_{i = 1}^{n} {\,\beta_{i} } X_{i} \, + \,\sum\nolimits_{i = 1}^{n} {\beta_{ii} X_{i}^{2} \, + \,\sum\nolimits_{i = 1}^{n} {\sum\nolimits_{j = 1}^{i - 1} {\beta_{ij} X_{i} X_{j} } } } , $$

where i, j, n, Y, X and β_0_ are the linear coefficient, quadratic coefficient, the number of independent factors, the response factor (biodiesel yield), the independent factor and the regression coefficient, respectively.

### Biodiesel characteristics

The biodiesel characteristics are measured by the following devices/techniques.

#### Fatty acid methyl ester content

To reveal the fatty acid methyl ester (FAME) composition of the biodiesel, a gas chromatograph 5973 (GC, Agilent Technologies) with a VF-1 (Agilent Technologies) capillary column (30 m × 0.25 mm × 0.25 μm) was utilized.

#### Kinematic viscosity

A Canon-Fenske Routine Viscometer (Canon USA Inc., State College, PA) was utilized to measure the viscosity of the biodiesel sample based on the ASTM D 92–85 method [55].

#### Density

The ASTM D 4052–91 was applied to estimate the density of biodiesel. The sample was poured into a graduated cylinder (100 ml) at a specific temperature (25 °C) and a hydrometer was used to determine the sample gravity [56].

#### Acid value

The KS M ISO 6618 (Korean standard association) was used to measure acid value. Initially, 10 g of the sample was poured into a flask with a 250-ml capacity. Afterward, 100 mL of ethanol and ether solution with a volume ratio of 1:2 was mixed with three drops of phenolphthalein and injected into the sample. The solution was titrated against 0.1 N KOH solution. The endpoint was determined based on pink color persistency (10 s). The acid value could be obtained by Eq. (5) as follows [57]:

5 $$AV=\frac{A\times N\times 5.611}{W},$$

where AV represents the acid value (mg KOH/g), A stands for the consumption of N/10 KOH (mL), N represents the titer of N/10 KOH (-), and W stands for the sample volume (mL).

#### Saponification value

Saponification value refers to short chains in alkyl groups of biodiesel fatty acids [56]. To measure the saponification value (SV), AOCS CD3 titration method was used. 1 g of mutton bone biodiesel was mixed with 25 ml of 0.5 N KOH solution. Besides, 4 ml of ethanol and ether solution with a volume ratio of 1:2 solution was introduced to the sample. The mixture is refluxed for 30 min. After cooling to 25 °C, a few drops of phenolphthalein were added to the solution as pH indicator and the endpoint was signaled by the appearance of the pink color. The solution was titrated in the presence of 0.5 N HCI solution till the pink color was eliminated. Besides, a blank titration on the water was done in the same quantity of KOH solution under the same condition and time. The saponification value (SV) could be calculated as follows [56]:

6 $$SV=56.1\times \frac{N\times \left({V}_{a}-{V}_{b}\right)}{M},$$

where M is the weight of biodiesel (g), V_a_ is the HCl volume Volume (ml) used in the test, V_b_ is the HCl volume (ml) used in blank and N is the Normality of HCL.

#### Iodine value

The level of unsaturation of biodiesel is evaluated by calculating the iodine value. One gram of biodiesel was dissolved in 10 ml of chloroform. Besides, 25 ml of Hanus solution was settled for 30 min in a dark space. Afterward, 10 ml of 15% KI (15 g KI dissolved in 100 ml) and the Hanus solution were added to the solution (biodiesel and chloroform). The prepared solution was titrated with 0.1 N Na_2_S_2_O_3_ until the yellow color of the solution turned into a transparent yellow color [58]. The iodine value could be calculated by Eq. (7):

7 $$IV=\frac{{M}_{1}\times \left({V}_{b}-{V}_{t}\right)}{{M}_{2}},$$

where IV is iodine value (g I_2_/100 g amostra, V_t_ is Na_2_S_2_O_3_ solution volume (mL) for the sample, V_b_ is Na_2_S_2_O_3_ solution volume for the blank (mL), M_1_ is mass of the biodiesel sample (g) and M_2_ is the molar mass of iodine.

#### Higher heating value

The empirical equations were used to determine the higher heating value (HHV). An increase in IV leads to a decrease in the heat content of a sample. Thus, Eq. (8) could be used to calculate the HHV (MJ/kg) of the biodiesel [59]:

8 $$HHV=49.43-\left[\frac{0.041\times SV+0.015\times IV}{{M}_{2}}\right].$$

#### Cetane number

The diesel quality testing by ASTM D613 was used to determine the cetane number (CN). The cetane number could be correlated as a function of IV and SN [60]:

9 $$CN=46.3+\left(\frac{5458}{SV}\right)-0.225\times IV.$$

## Results and discussion

### Response surface methodology

The effect of M/O, catalyst concentration and catalyst type on biodiesel yield was studied by using five-level three-factor CCD. Table 2 lists the input variables, the coded values and the experimental results for biodiesel yield introduced to Design-Expert software.

**Table 2:** Input variables, the coded values and the experimental results for biodiesel yield

Run	M/O (X_1_)	Catalyst concentration ((*wt* %) X_2_)	Catalyst type (X_3_)	Yield (%)
1	2	0	− 1	57.78
2	0	0	1	76.90
3	0	0	− 1	80.53
4	− 1	− 1	− 1	87.93
5	0	− 2	1	59.76
6	2	0	1	87.62
7	0	2	1	86.87
8	0	2	− 1	54.61
9	1	− 1	1	78.46
10	− 1	1	1	72.32
11	− 2	0	1	66.92
12	1	− 1	− 1	77.23
13	− 1	-1	1	71.70
14	0	0	1	78.35
15	0	0	− 1	79.12
16	1	1	1	89.74
17	− 1	1	− 1	72.32
18	− 2	0	− 1	82.72
19	0	− 2	− 1	81.14
20	1	1	− 1	63.43

#### Evaluation of regression model for the mutton biodiesel yield

The coefficients of the second-order polynomial equation (Eq. (4)) were determined and their significance was tested based on the regression analysis. The fitting model supported the significance of three linear coefficients (X_1_, X_2_, X_3_), three quadratic coefficients (X^2^_1_; X^2^_2_; X^2^_3_), and three cross-product coefficients (X_1_X_2_, X_1_X_3_, X_2_X_3_) (Tables 1, 2). The results of the analysis of variance (ANOVA) for the response surface model are listed in Table 3.

**Table 3:** Results of the analysis of variance (ANOVA) for mutton bone biodiesel

Source	Sum of squares	DF	Mean square	*F*-Value	*P* value Prob > *F*
Model	1914.58	8	239.32	23.07	0.0001
Residual	114.12	11	10.37		
Lack of fit	112.08	9	12.45	12.22	0.0779
Pure error	2.04	2	1.02		
Cor. total	2028.72	19			

Moreover, the model coefficients are examined. As shown in Table 3, the p-values of the linear coefficients are major compared to the other terms. Still, to diminish error, all the coefficients were taken into account. Based on Table 3, a small deficiency in fitting was observed; the model represents the appropriate relationship between the parameters. It was found that the X_3_ (linear effect of catalyst type), ${X}_{1}^{2}$ (quadratic effect of M/O), ${X}_{2}^{2}$ (quadratic effect of catalyst concentration), X_1_X_3_ (the interaction effect of M/O and catalyst type) and X_2_X_3_ (the interaction effect of catalyst concentration and catalyst type) have a significant effect on the biodiesel yield. The final estimate response model equation (based on the experimental data) for mutton biodiesel is as follows:

10 $$Y=79.41-0.16{X}_{1}-0.68{X}_{2}+1.59{X}_{3}+5.63{X}_{1}\times {X}_{3}+6.19{X}_{2}\times {X}_{3}-1.33{X}_{1}^{2}-{2.12X}_{2}^{2},$$

where Y represents the response factor, i.e., FAME content (% (w/w)) and X_1_, X_2_, and X_3_ stand for the quantities of the independent variables as indicated in Table 2. The coefficient estimates and probability values of the model are listed in Table 4.

**Table 4:** The coefficient estimates and significance of the response surface model

Factor	Coefficient estimate	Standard error	95% CI low	95% CI high	VIF
Intercept	79.41	1.36	76.41	82.40	
X_1_	− 0.16	0.66	− 1.61	1.28	1
X_2_	− 0.68	0.66	− 2.13	0.77	1
X_3_	1.59	0.72	0.0062	3.18	1
X^2^_1_	− 1.33	0.56	− 2.56	− 0.095	1.3
X^2^_2_	− 2.12	0.56	− 3.35	0.89	1.3
X_1_X_2_	1.56	1.14	− 0.95	4.06	1
X_1_X_3_	5.63	0.66	4.18	7.07	1
X_2_X_3_	6.19	0.66	4.74	7.64	1

#### Model accuracy check

According to the ANOVA results, the quadratic polynomial model could demonstrate the actual relationship of FAME yield and the model variables. In Table 5, the statistical summary of variance analysis for the proposed model is shown. The coefficients of determination (R^2^ = 0.9437 and R^2^_adj_ = 0.9028) for the obtained correlation are near unity, which shows the excellent significance of the proposed model.

**Table 5:** Statistical summary of variance analysis for the proposed model

Source	Amount (%)
Std.Dev	3.22
Mean	75.27
C.V.%	4.28
PRESS	423.73
− 2Log likelihood	91.59
R^2^	94.37
R_adj_^2^	90.28
R^2^_pred_	79.11
Adeq precision	15.961

**Fig. 1:**
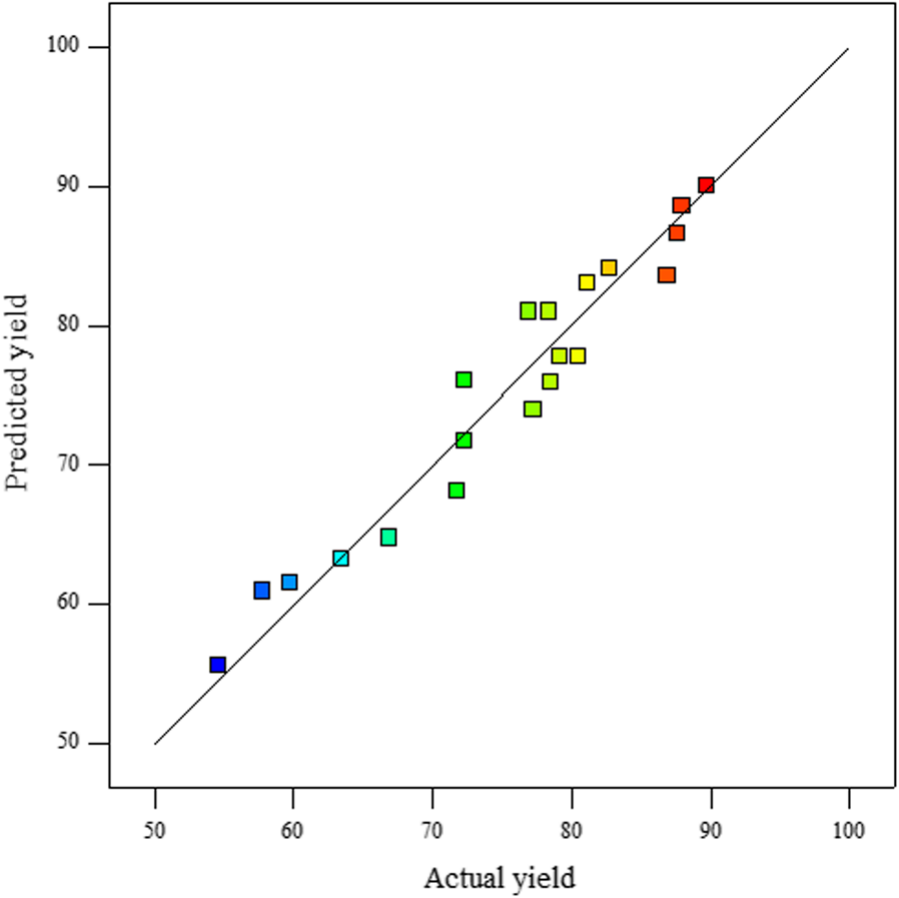
The predicted versus observed values for biodiesel yield (R^2^ = 0.9437 and R.^2^_adj_ = 0.9028)

Figure 1 illustrates a unit slope line that shows the zero deviation between the predicted and observed values. As can be seen, all the points are close to the line of the perfect fit. The figure approves the model’s accuracy. Therefore, the proposed correlation (Eq. (10)), is capable of predicting experimental outcomes.

### Effect of process variables on biodiesel synthesis

**Fig. 2:**
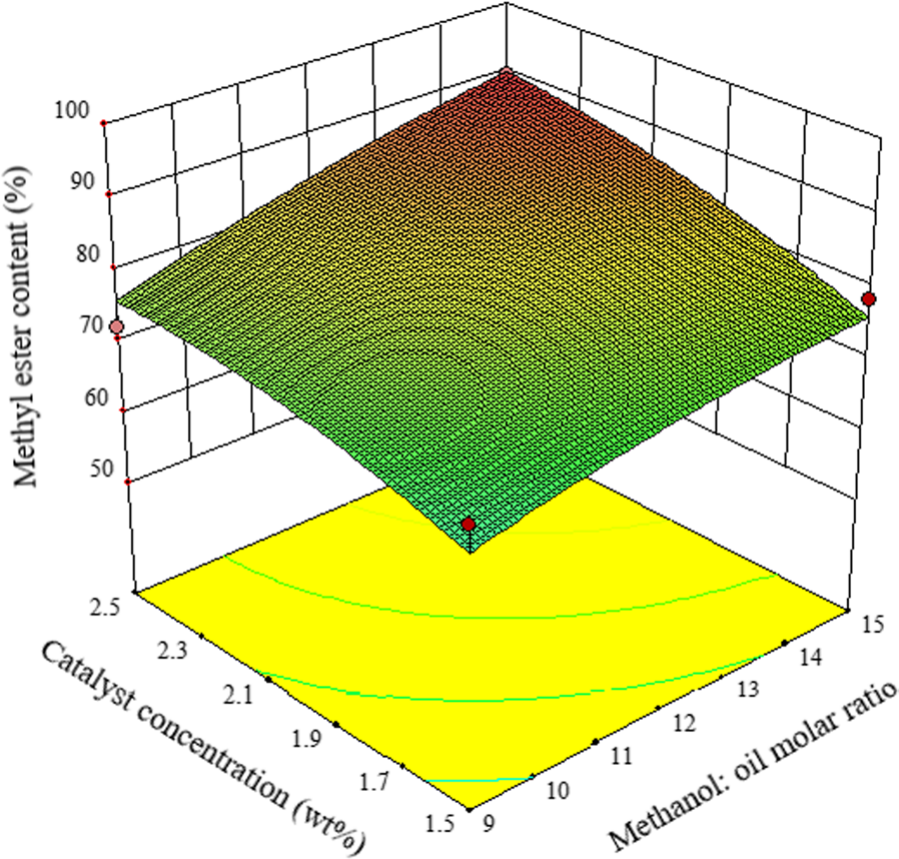
The effect of M/O, KOH concentration and their reciprocal interaction on mutton bone biodiesel synthesis at 60 °C, 3 h, and 500 rpm

Figure 2 illustrates the effect of M/O, KOH concentration and their reciprocal interaction on mutton bone biodiesel synthesis. The biodiesel yield is moderately influenced by the M/O at low catalyst concentration. This could be happen as increasing in the catalyst concentration could enhance the rate of the unwanted saponification reaction. On the other hand, at high catalyst concentration, a rise in M/O elevates the rate of transesterification reaction and compensates the effect of saponification reaction. Therefore, the impact of M/O is considerable at high catalyst concentration. The maximum acquisition of FAME content is achieved with a high M/O and 2.5 *wt*% catalyst concentration.

**Fig. 3:**
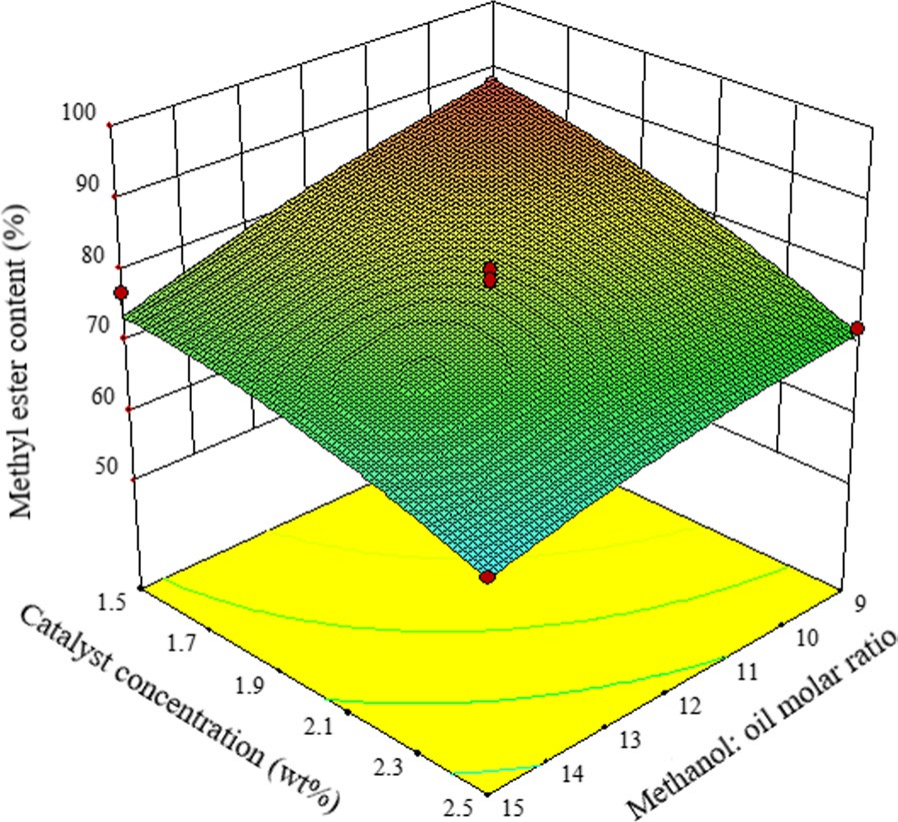
The effect of NaOH catalyst amount, M/O, and their reciprocal interaction on mutton bone biodiesel synthesis at 60 °C, 3 h, and 500 rpm

The influence of NaOH catalyst concentration and M/O on mutton bone biodiesel synthesis is shown in Fig. 3. Similar trend was observed for NaOH catalyst. The FAME content increases with a decrease in catalyst concentration and M/O. The highest content of methyl ester is achieved with a catalysis level of 1.5% (*w*/*w*) and M/O of 1:9.

**Fig. 4:**
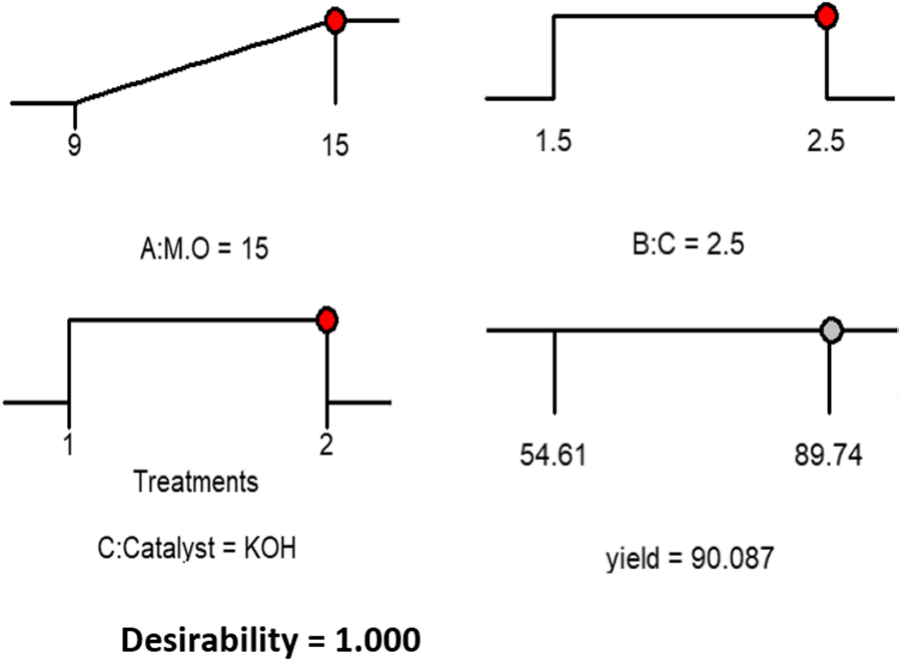
Desirability ramp of yield at 60 °C, 3 h, and 500 rpm

By considering the desirability function approach, the optimum value of the response surface, catalyst concentration, and M/O was obtained as presented in Fig. 4. The results approved that the maximum yield of 90.087% could be obtained with KOH catalyst concentration of 2.5 *wt*%; and M/O of 15:1.

To check the validity of the results obtained in Fig. 4, an experiment was set under these conditions. The experimental yield of mutton bone biodiesel was estimated as 90.0524%, which was in good consistency with the desirability function result (90.087%).

(In each figure, more explanation and description are necessary. The current version only lists what can be shown.)

### Characterization of the biodiesel sample

**Fig. 5:**
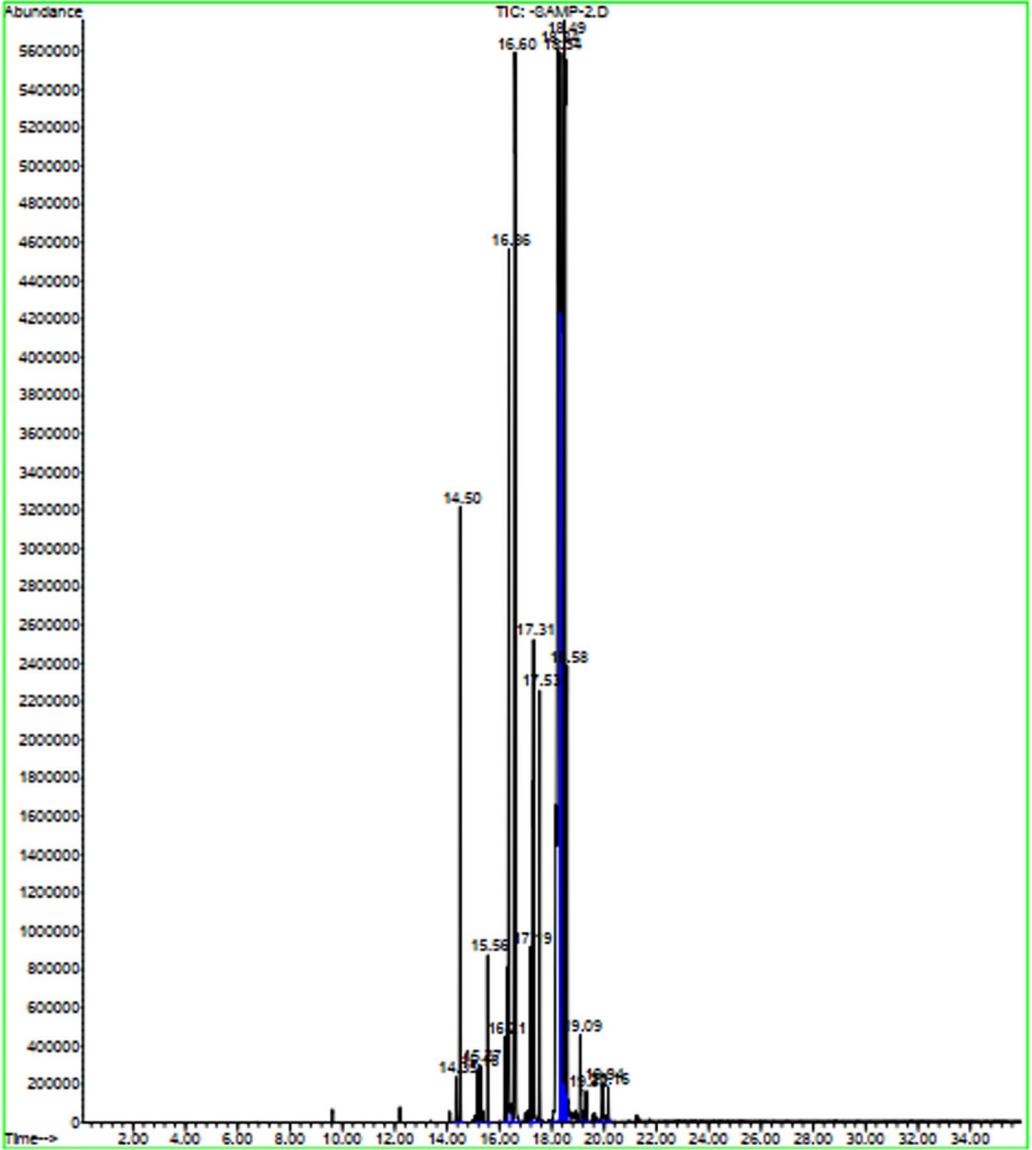
The GC/MS chromatogram analysis of mutton bone biodiesel

The analyzing methods for evaluating the characterization of the mutton bone biodiesel sample are previously discussed in Sect. 2–5. The GC/MS chromatogram analysis of biodiesel is shown in Fig. 5. The results indicate that biodiesel contains a variety of fatty acid methyl esters from C14 to C20, which include mostly palmitic acid (C16:0), stearic acid (C18:0) and oleic acid (C18:1). The FAME composition of the produced biodiesel from mutton bone oil is listed in Table 6. To make comparison possible, the FAME composition of the biodiesels generated from soybean oil, lard oil, chicken fat oil, and mutton fat oil are also listed in this table. It could be seen that the produced biodiesels from animal wastes are high in oleic acid (22–45%), palmitic acid (~ 17–23%) and stearic acid (~ 6–27%). However, the biodiesel generated from plant source lipid (soybean oil) most has Linoleic fatty acid. Furthermore, as a result of Table 6 and the analysis of the previous literature studies, the mutton bone biodiesel and mutton fat biodiesel feature notably higher stearic contents in comparison to chicken fat biodiesel.

**Table 6:** The comparison of the FA composition (*wt* %) of the mutton bone biodiesel with the other biodiesels

FA	Chemical structure	FAME composition (wt)%					Type of fatty acid
		Biodiesel from soybean oil [16]	Biodiesel from lard oil [16]	Biodiesel from chicken fat oil [61]	Biodiesel from mutton fat oil [61]	Biodiesel from mutton bone oil	
Myristic	C14:0	0	1.3	0.2286	0.7879	3.78	Saturated
Pentadecanoic	C15:0	0	0	–	–	1.67	Saturated
Palmitic acid	C16:0	10.6	23.5	24.654	28.1036	17.81	Saturated
Palmitoleic acid	C16:1	0	2.6	6.9231	0.4226	6.92	Unsaturated
Heptadecanoic	C17:0	0	0.4	0.1419	0.1387	3.4	Saturated
Stearic acid	C18:0	4.6	13.5	6.2515	27.1957	16.28	Saturated
Oleic acid	C18:1	22.1	41.7	45.1812	31.2798	41.12	Unsaturated
Linoleic acid	C18:2	54.2	10.7	12.5832	1.5957	2.62	Unsaturated
Nonadecylic acid	C19:0	–	–	–	–	0.91	Saturated
Arachidic acid	C20:0	–	–	0.0992	0.6075	0.65	Saturated

The comparison between the standard values with the experimental results is also tabulated in Table 7. The following 5 features admit the engine's good performance of biodiesel fuel prepared from mutton bone oil:

1. Flashpoint is the temperature at that a flame appears when the fuel is exposed to fire. This is a key characteristic of fuel in terms of the safety of storing and shipping fuel. This parameter is a function of fuel’s volatility, which is a key factor for engine start and warming function [67]. As listed in Table 7, the pure biodiesel obtained from mutton fat has a higher flashpoint compared to diesel. Therefore, biodiesel is considerably safer than diesel for storage. A low flashpoint of liquid fuel may inhibit auto explosion and risks at elevated temperatures.
2. Another key characteristic of fuel is its viscosity which influences the flow behavior. In the case of mutton bone biodiesel, the kinematic viscosity was higher than that of the ASTM standard (Table 7). The higher clearly, the kinematic viscosity values change depending on the type of FAME. For instance, higher quantities of saturated FAs along with bigger carbon chains lead to an increase in kinematic viscosity [68].
3. The presence of unsaturated FAs in biodiesel is needed to a specific extent to ensure no fuel solidification [67]. On the other hand, the high level of unsaturated FAs may lead to the formation of irreversible polymerized condensation products. As listed in Table 7, the iodine value of the obtained biodiesel was 36.54 grI_2_/100 ml of oil. The maximum iodine value of 120 grI_2_/100 ml was advised by EN-14214 [60], which admits the good performance of the produced biodiesel as a fuel.
4. The heating value referred to the enthalpy releases during the perfect ignition of fuel at constant volume. The greater the heating value of the fuel, the lesser the fuel volume needed to achieve the same power output for an engine [69]. It has been reported that the higher heating values (HHVs) of biodiesels are in the range of 39–41 MG/kg, which is moderately less than those of gasoline (46 MJ/kg), petrodiesel (43 MJ/kg), or petroleum (42 MK/kg) [56]. Therefore, the HHV of the produced biodiesel (40.0265 MJ/kg) gives an appropriate value.
5. In general, a higher CN value for diesel fuel means a shorter ignition delay and combustion duration, fewer knocking, and fewer nitrogen oxides (NO_x_) [70, 71]. The CN value for the mutton bone biodiesel (63.37) is higher than that of the diesel (47–55) (Table 7). This makes the mutton bone biodiesel more attractive than diesel fuel. Furthermore, it could be used as a cetane enhancer in petroleum diesel fuels.

**Table 7:** Fuel properties of biodiesel from mutton bone oil

Properties	Unit	EN-14214 [62, 60, 56]	ASTM-D6751 [60, 63–65]	ASTM No. 2D diesel [53, 56, 66]	Biodiesel from mutton bone oil
Density (@ 25 °C)	Kg/m^3^	860–900	–	840–860	875
Kinematic viscosity (@ 40 °C)	mm^2^/s	3.5–5	1.9–6	1.9–3.8	5.26
Flash point	°C	120 min	130 min	70	168
Acid value	mg KOH/g	0.5 max	0.8 max	0.5	0.673
Saponification value	mg KOH/g	–	370 max	–	215.985
Iodine value	gr I_2_/100	120 max	–	–	36.54
Cetane index	–	51 min	47 min	47–55	63.37
Higher heating value	MJ/kg	35	37.37–40.168	43.3–46.7	40.03

## Modeling of methanol + mutton bone fat mixture for biodiesel production in a membrane reactor system

In this part, a membrane system with a recycle stream is proposed to compensate for the long residence time of the biodiesel production process from methanol + mutton bone fat mixture. The recycled stream could improve the transesterification rate and product yield. On the other hand, the membrane system is applied for purifying biodiesel.

### Determination of the reaction kinetics

The following reaction could be considered for biodiesel production from methanol + mutton bone fat mixture [72, 73]:

11 $$TG+3M\to P+G,$$

where [TG], [M], [P] and [G] denote the triglyceride, methanol, FAME and glycerol concentrations.

The impact of ultrasonic waves on reactants (TG and M) are not the same. Therefore, Eq. 12 is proposed for the rate equation to consider individual reaction rate orders [72]:

12 $${r}_{P}=\frac{d\left[P\right]}{dt}=k{\left[TG\right]}^{\alpha }{\left[M\right]}^{\beta }.$$

The linearization equation is obtained from Eq. (12):

13 $$ln\left({r}_{P}\right)=ln\left(k\right)+\alpha \times ln\left(\left[TG\right]\right)+\beta \times ln\left(\left[M\right]\right).$$

Experimental data were curve-fitted in Eq. 13. The results are shown in Table 8.

**Table 8:** Reaction kinetic constants for the proposed model (T = 60 °C)

Parameter	Value
*k* (*lit/(mol.min)*)	2.415 × 10^–2^
*α*	1
*β*	1

### Mathematical modeling of the membrane reactor system

**Fig. 6:**
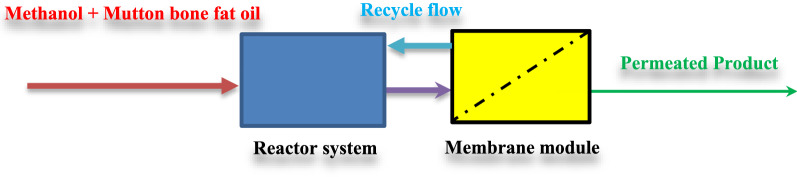
Schematic of the membrane reactor system used for the biodiesel production

Figure 6 shows a membrane system proposed by biodiesel production. The reactants including methanol + mutton bone fat oil mixture are fed to the fully mixed reactor system. The products are then directed to a membrane system. The biodiesel (FAME) is permeated through the membrane and the retentate is recycled to the reactor. Therefore, the biodiesel purification and reaction rate enhancement will take place simultaneously. In this study, a Filtanium ceramic membrane (TAMI, Nyons, France) constructed of a titanium oxide support and the active layer was selected based on the work prepared by Cao et al. [48].

For finding the kinetics of mutton bone oil biodiesel with an ultrasonic bath the following assumptions are considered:

- 1-The recycled stream is fully mixed which is equivalent to the statement that all the properties of this stream (e.g., temperature and concentration are uniforms.
- 2-The feed mass flow rate is assumed to equal to the product mass flowrate.
- 3-Triglyceride is not presented in the reactor outlet stream.
- 4-Two distinct phases were formed: methanol, glycerol and FAME are presented in a single mobile phase and triglyceride is formed in another phase in the reactor.
- 5-Only FAME can permeate through the membrane.

Equation (14) could be obtained by the mentioned assumptions [49]:

14 $$ \mathop {\text{V}}\limits^{.}_{{{\varvec{out}}}} = \frac{{_{{\mathop {\text{m}}\limits^{.}_{{{\varvec{Total}} - {\varvec{in}}}} }} }}{{\rho_{out} }} = \frac{{F_{Methanol - in} \times MW_{Methanol} + F_{TG - in} \times MW_{TG} }}{{\rho_{out} }}. $$

In Eq. (14), $$\mathop {\text{V}}\limits^{.}_{out}$$$$,\boldsymbol{ }\boldsymbol{ }\boldsymbol{ },\boldsymbol{ }{F}_{Methanol-in},\boldsymbol{ }{F}_{TG-in}\boldsymbol{ }\mathrm{and}\boldsymbol{ }{MW}_{TG},\boldsymbol{ }{\rho }_{out}$$ are permeate flow rate of the (lit/min), mass flow rate of the feedstock, methanol molar rate in the feed stream (mol/min), Triglyceride molar rate in the feed stream (mol/min), Triglyceride molecular weight (g/mol) and product density (g/mlit), respectively.

The components molar balances are illustrated in the following:

15 $$\frac{d(Methanol)}{dt}={F}_{Methanol-in}+{r}_{Methanol}{\times V}_{0},$$

16 $$\frac{d(TG)}{dt}={F}_{TG-in}+{r}_{TG}\times {V}_{0},$$

17 $$\frac{d(FAME)}{dt}={r}_{FAME}{\times V}_{0}-{F}_{FAME-out},$$

18 $$\frac{d(G)}{dt}={r}_{G}{\times V}_{0,}$$

where

19 $$ F_{FAME - out} = \left[ {FAME} \right]_{out} \mathop {\text{V}}\limits^{.} \times_{out} . $$

The volume of the mobile phase (Vmobile) can be calculated by Eq. (20):

20 $${V}_{mobile}={V}_{0}-\frac{\left[TG\right]\times {V}_{0}\times {MW}_{TG}}{{\rho }_{TG}}.$$

The concentration of the FAME in the mobile phase passing through the membrane could be obtained from Eq. 21:

21 $${\left[FAME\right]}_{out}=\frac{{V}_{0}\times \left[FAME\right]}{{V}_{mobile}}=\frac{\left[FAME\right]}{\left(1-\frac{\left[TG\right]\times {MW}_{TG}}{{\rho }_{TG}}\right)}.$$

In Eqs. (15) to (21), V_0_, [x], r, t, F_x-in_ and F_x-out_ are reactor volume (lit), concentration of component x (mol/lit), reaction rate (mol/(lit.min)), time (t), flowrate of component x in feed and product streams (mol/min), respectively. The modeling was performed by MATLAB 2016a software. The modified Rosenbrock method (ode23s) was used to solve the set of stiff ordinary differential equations (ODEs).

#### Model validation

**Fig. 7:**
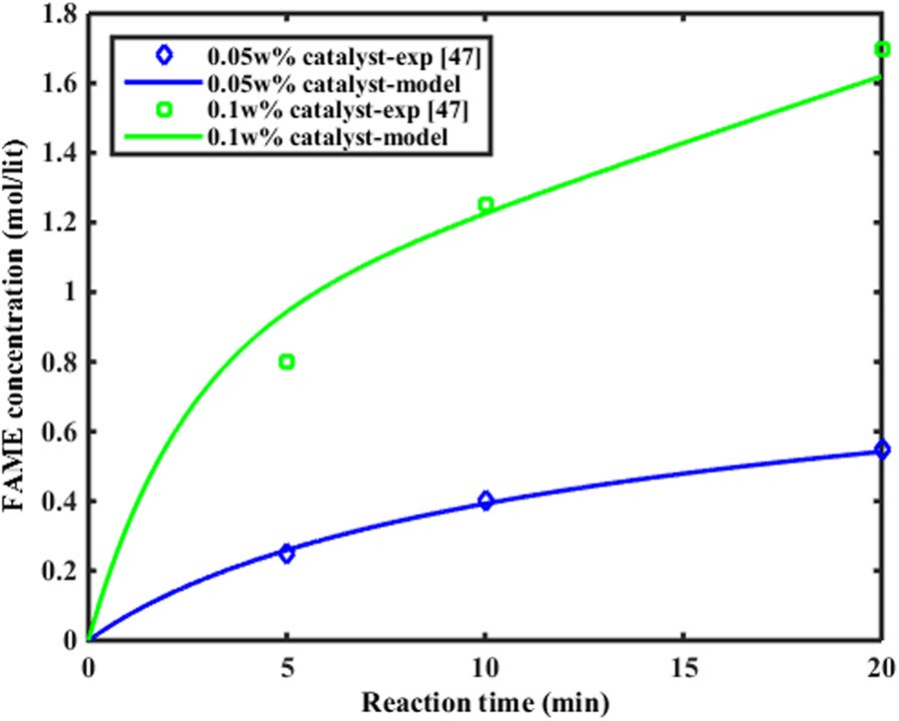
The comparison of experimental and calculated FAME concentration variation in the membrane reactor

At first, the mathematical modeling results were compared with experimental data obtained from Cao et al.’s work [48]. They produced biodiesel from canola oil using a 6-L membrane reactor system [48]. Figure 7 shows the comparison of experimental and calculated FAME concentration variation in the membrane reactor.

#### The proposed membrane system for biodiesel generation from methanol + mutton bone fat mixture

Table 9 presents the system specifications for the membrane system. The system conditions are similar to those used for experimental tests.

**Table 9:** The operating parameters of the membrane reactor system

Parameter	Value
Feed molar flowrate of TG (mole/min)	0.6
Feed molar flowrate of methanol (mole/min)	8.3
The initial concentration of TG (mole/lit)	0.69
The initial concentration of methanol (mole/lit)	9.84
Reactor volume (lit)	80
Reaction temperature (°C)	60

**Fig. 8:**
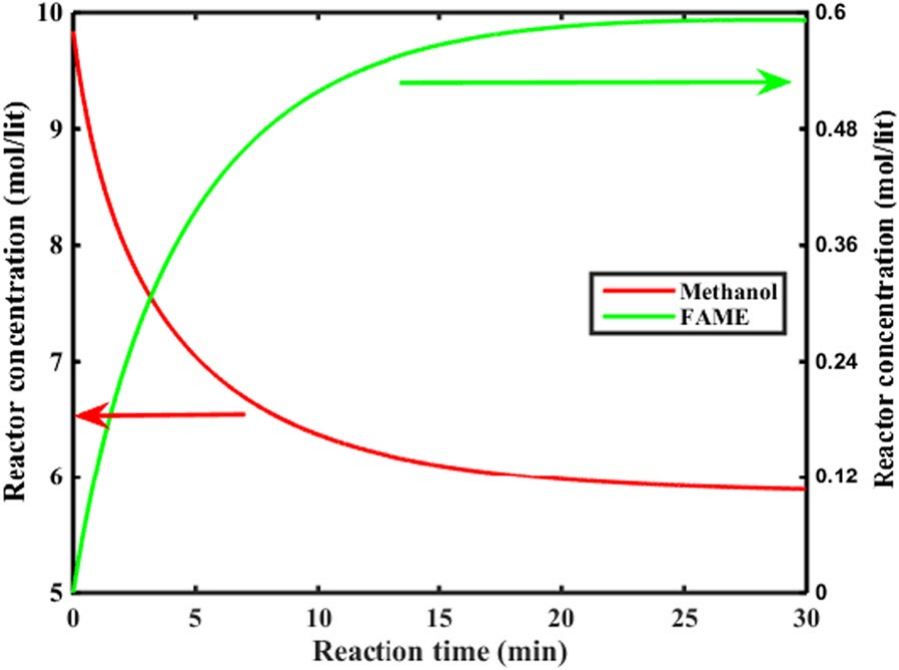
Methanol and FAME concentrations at reactor outlet, T = 60 °C, reactor volume = 80 lit

The membrane system performance for biodiesel production is investigated. Figure 8 shows the methanol and FAME concentrations in the membrane system at optimum condition (Fig. 4). As can be seen, after 30 min, a significant amount of FAME has been produced, which indicates the high efficiency of the proposed membrane system. It means that the reaction time in the membrane system (30 min) is 6 times shorter than the conventional method (3 h).

**Fig. 9:**
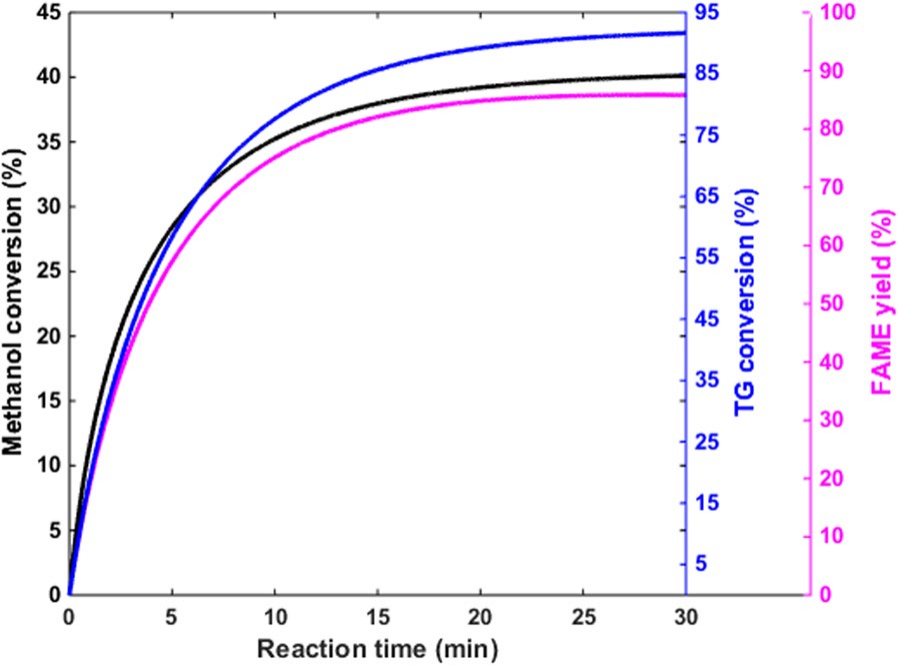
Methanol conversion, TG conversion and FAME yield

The biodiesel yield, the TG and the methanol conversion rates are shown in Fig. 9. A comparison between the biodiesel yield with Fig. 4 shows that the yield of the proposed system is equivalent to a single batch reactor with a reaction time of 3 h. Therefore, the membrane system has significantly improved the reaction rate.

## Conclusions

The application of methanol for biodiesel production from mutton bone was examined experimentally. Two homogenous catalysts (KOH and NaOH) are used in an ultrasound-assisted system to perform the transesterification process. A response surface design methodology was assisted to evaluate the effects of M/O, type of alkali catalyst and catalyst concentration on biodiesel yield at the frequency of 25 kHz and temperature of 60 °C. Optimization of the process of biodiesel production was achieved by five-level-three-factors CCD using RSM. A second-order model was found to calculate the mutton bone yield as a function of the process variable. The optimum conditions were obtained as a methanol/oil of 15:1 and KOH concentration of 2.5 *wt*%. Under these conditions, the conversion rate exceeds 90% in three hours.

The analysis of density, acid value, saponification value, iodine value, kinematic viscosity and combustion properties showed the good performance of mutton bone biofuel generated through methanol in comparison with fossil fuels. The obtained biodiesel met the quality standards of American Communities. Finally, a mathematical model of biodiesel production in a membrane system is developed. The reaction rate constant is calculated as a function of ultrasonic frequency. Compared with the conventional method, the membrane system has significantly improved the reaction rate.

## Data Availability

No data are associated with this article.
